# Principal components of tau positron emission tomography and longitudinal tau accumulation in Alzheimer’s disease

**DOI:** 10.1186/s13195-020-00685-4

**Published:** 2020-09-23

**Authors:** Hanna Cho, Min Seok Baek, Hye Sun Lee, Jae Hoon Lee, Young Hoon Ryu, Chul Hyoung Lyoo

**Affiliations:** 1 Department of Neurology, Gangnam Severance Hospital, Yonsei University College of Medicine, 20 Eonjuro 63-gil, Gangnam-gu, Seoul, South Korea; 2 Biostatistics Collaboration Unit, Yonsei University College of Medicine, Seoul, South Korea; 3 Department of Nuclear Medicine, Gangnam Severance Hospital, Yonsei University College of Medicine, 211 Eonjuro, Gangnam-gu, Seoul, South Korea

**Keywords:** Alzheimer’s disease, Positron emission tomography, Tau, ^18^F-flortaucipir

## Abstract

**Background:**

We aimed to investigate the clinical correlates of principal components (PCs) of tau positron emission tomography (PET) and their predictability for longitudinal changes in tau accumulation in Alzheimer’s disease (AD).

**Methods:**

We enrolled 272 participants who underwent two PET scans [^18^F-flortaucipir for tau and ^18^F-florbetaben for amyloid-β (Aβ)], brain magnetic resonance imaging, and neuropsychological tests as baseline assessments. Among them, 187 participants underwent the same follow-up assessments after an average of 2 years. Using Aβ-positive AD dementia-specific PCs obtained from the baseline scans of 56 Aβ-positive patients with AD dementia, we determined the expression of the first two PCs (PC1 and PC2) in all participants. We assessed the correlation of PC expression with baseline clinical characteristics and tau accumulation rates. Moreover, we investigated the predictability of PCs for the longitudinal tau accumulation in training and test sets.

**Results:**

PC1 corresponded to the tau distribution pattern in AD, while the two PC2 extremes reflected the parietal or temporal predominance of tau distribution. PC1 expression increased with tau burden and decreased with cognitive impairment, while PC2 expression decreased with advanced age and visuospatial and attention function deterioration. The tau accumulation rate was positively correlated with PC1 expression (greater tau burden) and negatively correlated with PC2 expression (temporal predominance). A regression model using both PCs could predict longitudinal changes in the tau burden (intraclass correlation coefficient = 0.775, *R*^2^ = 0.456 in test set).

**Conclusions:**

PC analysis of tau PET could be useful for evaluating disease progression, characterizing the tau distribution pattern, and predicting longitudinal tau accumulation.

## Background

The recent development of tau-selective positron emission tomography (PET) radiotracers has facilitated research on neurodegenerative diseases [1]. ^18^F-flortaucipir, which is the most widely investigated radiotracer, selectively binds to abnormally hyperphosphorylated tau protein in postmortem tissue [2]; therefore, it enables in vivo visualization of tau pathology in Alzheimer’s disease (AD) [3, 4]. Patients with AD present with increased ^18^F-flortaucipir binding in the medial temporal cortex and association neocortices with relative sparing of the primary sensorimotor and visual cortices [4, 5]. Cortical ^18^F-flortaucipir binding is correlated with the degree of cortical atrophy and cognitive impairment degree [4, 5] and mirrors the pathological stage of AD [3]. Moreover, longitudinal changes in cortical uptake are reflective of the clinical progression [6]. Therefore, ^18^F-flortaucipir could be a surrogate biomarker for the progression of AD.

Principal component analysis (PCA) is a data-driven method for establishing patterns that explain variance by reducing the high dimensionality of image data [7]. This method has been used to identify the specific metabolic and amyloid-β (Aβ) accumulation patterns for AD and healthy aging [7–10]. Using PCA, we aimed to identify principal components (PCs) of ^18^F-flortaucipir PET in AD dementia. Thereby, we sought to find their clinical correlates and to investigate the predictability for the longitudinal changes in tau accumulation.

## Materials and methods

### Participants

From January 2015 to August 2017, we enrolled 176 patients at the Memory Disorder Clinic in Gangnam Severance Hospital and 96 healthy controls. We first enrolled patients with amnestic-type dementia with a probable AD diagnosis according to the National Institute of Neurological and Communicative Disorders and Stroke and the Alzheimer Disease and Related Disorders Association [11], as well as patients with a mild cognitive impairment (MCI) diagnosis according to Petersen’s criteria. The patients with AD dementia first presented with amnesia and did not present clinical features atypical for AD, including posterior cortical atrophy, logopenic aphasia, or frontal-variant AD. The healthy controls met the Christensen’s diagnostic criteria and underwent similar clinical and neuroimaging assessments [12]. The healthy controls presented normal cognition based on the neuropsychological test battery, no previous history of head trauma and neurological/psychiatric illness, and no brain magnetic resonance (MR) imaging abnormality except for the mild to moderate degree of white matter hyperintensities. For baseline assessments, all the participants underwent a clinical interview, neuropsychological test battery, genotyping for ApoE, and brain MR imaging, as well as ^18^F-flortaucipir and ^18^F-florbetaben PET imaging. Among the 272 participants, 187 participants underwent similar follow-up neuropsychological and neuroimaging assessments after a mean duration of 2.0 ± 0.3 years.

### Standard protocol approvals, registrations, and patient consent

This study was approved by the institutional review board of Gangnam Severance Hospital. We obtained written informed consent from all the participants. All procedures performed in this study were in accordance with the ethical standards of the 1964 Helsinki declaration and its later amendments.

### Neuropsychological tests

All the participants underwent the Seoul Neuropsychological Screening Battery (SNSB) [13]. Cognitive domain functions were measured using the scorable items as proposed by the SNSB developers [14]. To assess global cognitive function, we measured the mini-mental state examination scores, clinical dementia rating sum-of-boxes (CDR-SB) scores, and composite scores for five cognitive domains [14].

### Acquisition of PET and MR images

We performed ^18^F-flortaucipir and ^18^F-florbetaben PET on separate days. We acquired PET images using a Biograph mCT PET/CT scanner (Siemens Medical Solutions, Malvern, PA, USA) for 20 min at 80 and 90 min after ^18^F-flortaucipir and ^18^F-florbetaben injections, respectively. We reconstructed 3D PET images in 256 × 256 × 223 matrices with 1.591 × 1.591 × 1 mm voxel size using the ordered-subsets expectation-maximization algorithm after attenuation correction. Aβ-positivity was determined after agreement of two nuclear medicine specialists after visual assessment [15, 16]. T1-weighted brain MR images were acquired using a 3.0 Tesla MR scanner (Discovery MR750, GE Medical Systems, Milwaukee, WI) with 3D spoiled gradient-recalled sequences (repetition time = 8.28 ms, echo time = 1.6 to 11.0 ms, flip angle = 20°, 512 × 512 matrix, voxel spacing = 0.43 × 0.43 × 1 mm).

### Image processing steps

First, MR images were processed using FreeSurfer 5.3 (Massachusetts General Hospital, Harvard Medical School; http://surfer.nmr.mgh.harvard.edu) to obtain participant-specific volume-of-interest (VOI) images with the subcortical and cortical regions labeled as previously described [3]. PET images were co-registered to individual MR images within the FreeSurfer space (256 × 256 × 256 matrix with 1 mm isovoxels) and then corrected for partial volume effect (PVE) using regional mask images based on the region-based voxel-wise method [17]. Subsequently, we converted PVE-corrected PET images to standardized uptake value ratio (SUVR) images with the cerebellar crus median as a reference. We measured regional SUVR values by overlaying participant-specific VOI images on the obtained PVE-corrected PET images.

We used statistical parametric mapping 12 (SPM12, Wellcome Trust Centre for Neuroimaging, London, UK) and in-house software implemented in MATLAB 2015b (MathWorks, Natick, MA, USA) for spatial normalization and PCA. We spatially normalized inhomogeneity-corrected MR images to the in-house diffeomorphic anatomical registration using exponentiated lie algebra (DARTEL) template in 181 × 217 × 181 matrices with 1 mm isovoxels using the DARTEL toolbox in SPM12 [18]. Subsequently, we normalized the PVE-corrected SUVR images by applying flow fields normalizing gray and white matter. Finally, spatially normalized SUVR images were smoothed in a 3D space using a Gaussian kernel with 8 mm full-width half-maximum followed by PCA performance.

### Principal component analysis

For PCA, we used in-house software implemented in MATLAB, as previously described [8]. For all the participants, SUVR values of the gray matter voxels in the in-house atlas were first transferred to a row vector with 628,181 elements after demeaning the values [19]. To obtain Aβ-positive AD dementia-specific PCs, we created an *M* × *N* data matrix (*M* = number of patients, *N* = number of vector elements) using the 56 Aβ-positive patients with AD dementia. The data matrix was decomposed into three matrices through singular value decomposition; subsequently, we obtained an M × N matrix for Aβ-positive AD dementia-specific PCs. We converted row vectors for each PC into 3D volumes for visualization. Using these Aβ-positive AD dementia-specific PCs as a reference, we calculated the estimated component weights reflecting the expression of each reference PC in each gray matter voxel in all participants. Based on the PC1 and PC2 expression, we classified 114 Aβ-positive individuals into quartile groups.

### Statistical analysis

All statistical analyses were performed using SPSS 23 (IBM Corp., Armonk, NY, USA). Between-group comparisons of demographic data were performed using the one-way ANOVA test or independent *t* test for continuous variables and the chi-square test for categorical variables. We used Pearson’s correlation analysis to determine the correlation of PC expression with age and global cortical SUVR. Moreover, we used partial correlation analysis to determine the correlation between PC expression and cognitive functions with adjustments for age, years of education, sex, and the presence of ApoE ε4 allele. Partial correlation analysis was performed in two stages: the first involved a calculation of the standardized residuals of the linear regression analysis with the demographic data as independent variables, and the second involved Pearson’s correlation analysis between the standardized residuals for the two variables of interest. To investigate the association between PC expression and longitudinal tau accumulation, we first compared annual changes in the SUVR values between the quartile groups for PC1 and PC2 expression by using the general linear model with adjustments for age, years of education, sex, presence of ApoE ε4 allele, and baseline global cortical SUVR values. We used Bonferroni’s method to correct for multiple comparisons. Second, we performed partial correlation analysis between PC expression and annual changes in the SUVR values after adjusting for the same covariates. Regarding prediction of longitudinal changes in tau accumulation, we conducted linear regression analysis with the annual changes measured by the two tau PET studies as the dependent variables and PC1 expression/PC1 and PC2 expression, age, years of education, sex, and presence of ApoE ε4 allele as independent variables. To validate this model, we first divided the samples into two equal groups based on the date of the baseline scan. One group served as a training set to obtain the parameters for the regression equation, while the other group was used to calculate the predicted annual changes.

### Data availability statement

The data that support the findings of this study are available on request from the corresponding author. The data are not publicly available due to privacy restrictions.

## Results

### Demographic characteristics

Table 1 summarizes the demographic characteristics. Patients with AD dementia and MCI were older than healthy controls. Patients with AD dementia were less educated and more frequently presented the ApoE ε4 allele compared to the controls; moreover, they had longer disease duration than patients with MCI. There were significant between-group differences in the Aβ-positivity and test performances for global cognition and five cognitive domains.

**Table 1:** Demographic characteristics of the study participants

	CU	MCI	DEM
***n***	96	105	71
**Age (years)**	66.3 ± 9.5	71.1 ± 9.1^a^	74.4 ± 9.3^a^
**Sex (M to F)**	36: 60	41: 64	17: 54
**Education (years)**	11.9 ± 4.5	11.2 ± 4.5	9.8 ± 5.7^a^
**Duration (years)**	n.a.	2.3 ± 1.3	3.5 ± 1.5^b^
**ApoE ε4 genotype**	17/96 (18%)	30/105 (29%)	30/71 (42%)^a^
**Amyloid positivity**	9/96 (9%)	49/105 (47%)^a^	56/71 (79%)^ab^
**MMSE**	28.2 ± 1.8	25.7 ± 2.7^a^	19.1 ± 5.3^ab^
**CDR-SB**	0	1.5 ± 1.0^a^	5.0 ± 2.5^ab^
**Total cognition score**	183.2 ± 33.8	135.2 ± 33.6^a^	86.4 ± 34.7^ab^
**Memory**	73.3 ± 20.6	44.1 ± 17.9^a^	23.6 ± 13.9^ab^
**Language**	23.8 ± 3.1	20.5 ± 4.2^a^	16.2 ± 5.6^ab^
**Visuospatial**	33.1 ± 4.7	29.6 ± 7.2^a^	21.0 ± 11.5^ab^
**Frontal/executive**	42.1 ± 9.2	31.5 ± 10.3^a^	17.8 ± 9.3^ab^
**Attention**	10.9 ± 2.5	9.6 ± 2.7^a^	8.1 ± 2.6^ab^

*Abbreviations*: *CU* cognitively unimpaired, *DEM* Alzheimer type dementia, *MCI* mild cognitive impairment, *ApoE* apolipoprotein E, *MMSE* Mini-Mental State Examination, *CDR-SB* Clinical Dementia Rating sum-of-boxes, *n.a.* not applicable

^a^*P* < 0.05 for the comparison with CU

^b^*P* < 0.05 for the comparison with MCI

### Principal components and baseline characteristics

**Fig. 1:**
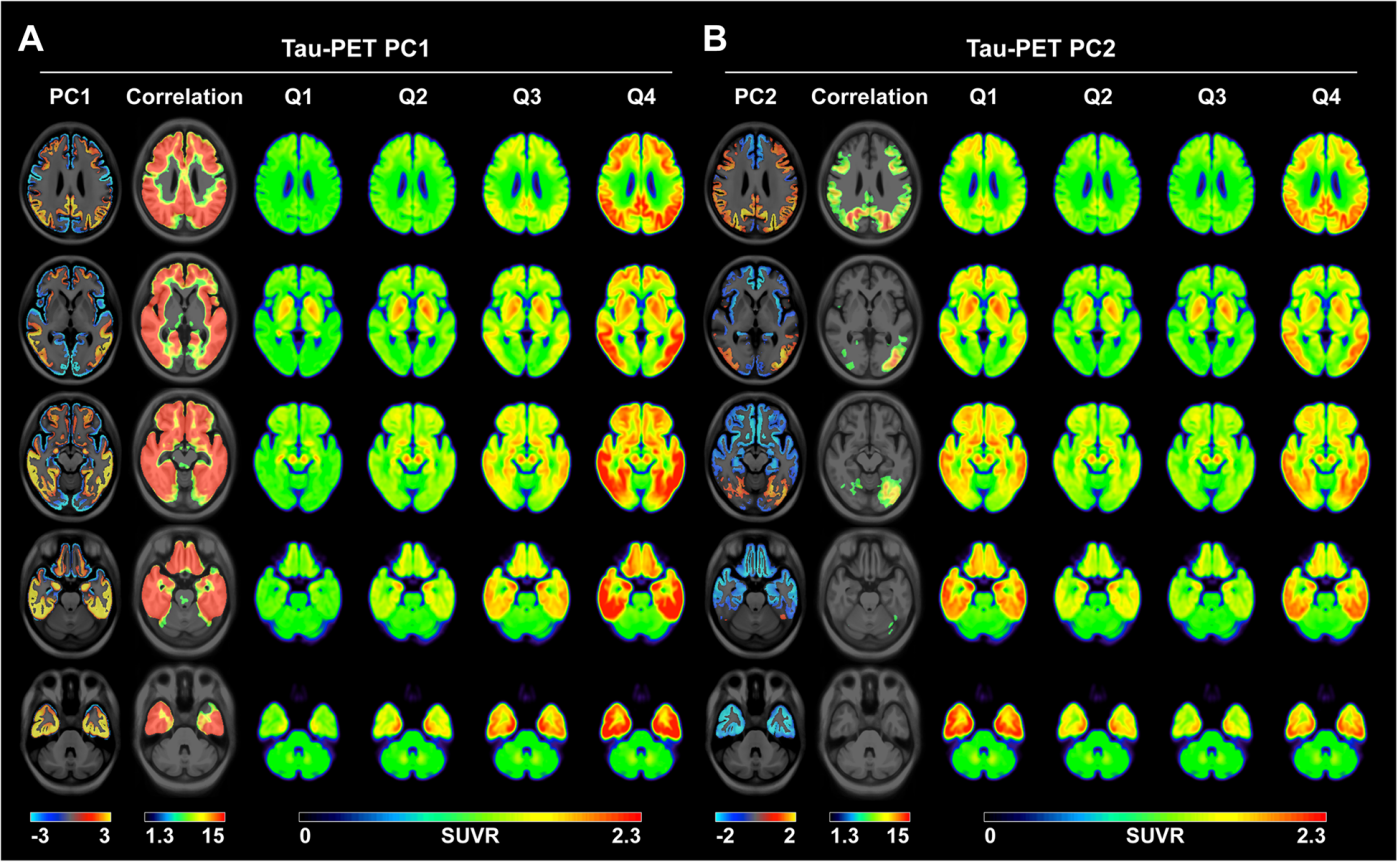
Voxel-wise maps for Aβ-positive AD dementia-specific PCs of tau PET and group-wise averaged tau PET images for each quartile of PC scores in 114 Aβ-positive individuals. Individual PC1 scores correlated with the tau uptake in the entire cortex and there was an increasing trend of cortical tau burden with the advancement of the PC1 quartile groups. Individual PC2 scores correlated with the tau uptake in the prefrontal, parietal, occipital, and posterior cingulate cortices. The lowest PC2 quartile group (Q1) showed a temporal predominance pattern while the highest PC2 quartile group (Q4) showed a parietal predominance pattern. Significance maps for the voxel-wise correlation analysis between the individual scores for the PCs and tau uptake are overlaid on the template. Only the voxels that survived after correcting for multiple comparisons with family-wise error (*P* < 0.05) are displayed. Color bars represent PCs (bidirectional cold and hot colors), −Log_10_*P* (rainbow color) and SUVR (rainbow color). PC, principal component; AD, Alzheimer’s disease; SUVR, standardized uptake value ratio; Qn, quartiles

The Aβ-positive AD dementia-specific PC1 pattern was very similar to the tau distribution and spreading pattern reported by previous postmortem and tau PET studies (Fig. 1a) [3–5, 20]. Group-wise averaged ^18^F-flortaucipir PET images for each PC1 score quartile in the 114 Aβ-positive individuals showed an increasing trend of cortical uptake in the more widespread cortex. Moreover, Aβ-positive AD dementia-specific PC2 showed two extreme ^18^F-flortaucipir uptake patterns, i.e., temporal and parietal predominance, which were reflected by the averaged images of the first and fourth quartile groups for PC2 expression in the 114 Aβ-positive individuals (Fig. 1b). PVE-uncorrected data also showed similar PC patterns (Additional file 1: Fig. S2).

**Fig. 2:**
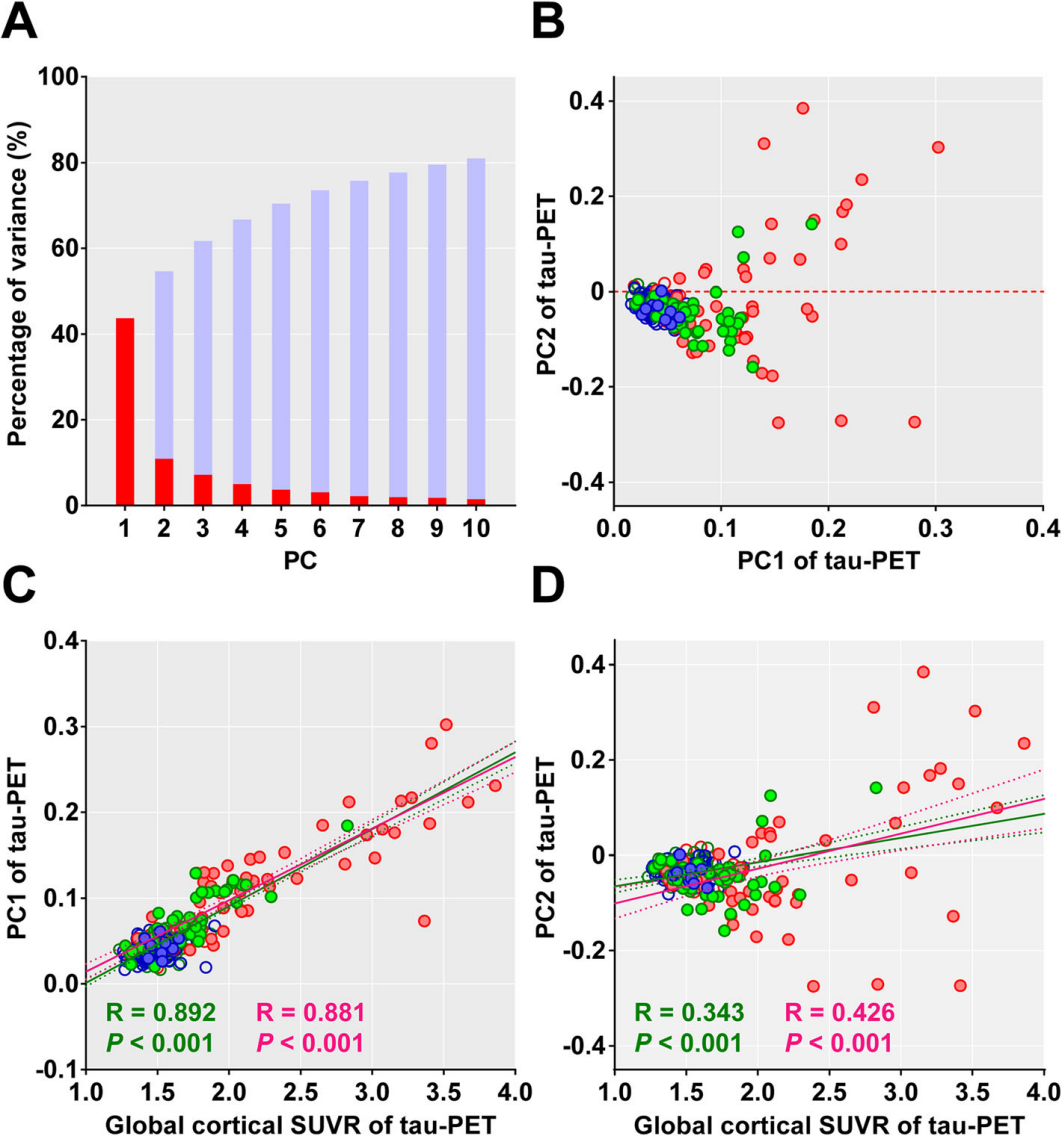
Expression of Aβ-positive AD dementia-specific PCs and global cortical tau burden. **a** The percentages of variance explained by the first 10 PCs (red bars) and their cumulative percentages (light blue bars). The first two PCs (PC1 and PC2) contributed to 44% and 11% of total variance, respectively. **b** Scatter plot of individual scores for PC1 and PC2 expression exhibited divergence of PC2 scores with increased PC1 scores. **c** PC1 scores clearly reflect the cortical tau burden measured by global cortical SUVR. **d** PC2 scores were likely to increase with the global cortical SUVR; however, there was a divergence of PC2 scores among individuals with a higher tau burden. The colors of the circles represent individual clinical status (blue = CU, green = MCI, and red = dementia), and the closed and open circles represent Aβ-positive and Aβ-negative individuals, respectively. Pearson’s correlation lines (solid), 95% confidence interval lines (dotted), correlation coefficients (*R*), and *P* values are presented in green color (all 272 individuals) or red color (114 Aβ-positive individuals). PC, principal component; AD, Alzheimer’s disease; MCI, mild cognitive impairment; CU, cognitively unimpaired

The first two Aβ-positive AD dementia-specific PCs explained 55% of the variances (PC1 = 44% and PC2 = 11%; Fig. 2a). There was a correlation between the expression of two PCs (*P* = 0.001, R = 0.200); however, there was a diverging trend of PC2 scores in individuals with higher PC1 scores (Fig. 2b). The global cortical SUVR values were correlated with both PC1 and PC2 scores; however, the correlation was much stronger with PC1 scores than with PC2 scores as expected, given the Aβ-positive AD dementia-specific PC patterns (Fig. 2c, d). Moreover, there was a correlation between the PC1 scores and global cortical SUVR values even in the Aβ-negative individuals (*P* < 0.001, *R* = 0.412). When we compared the regional SUVR values of the first and fourth quartile groups of PC2 expression, the fourth quartile group showed greater ^18^F-flortaucipir SUVR values in the sensorimotor, superior and inferior parietal, and precuneus cortices. Contrastingly, the first quartile group showed greater ^18^F-flortaucipir SUVR values in the entorhinal cortex and hippocampus (Additional file 1: Table S1).

**Fig. 3:**
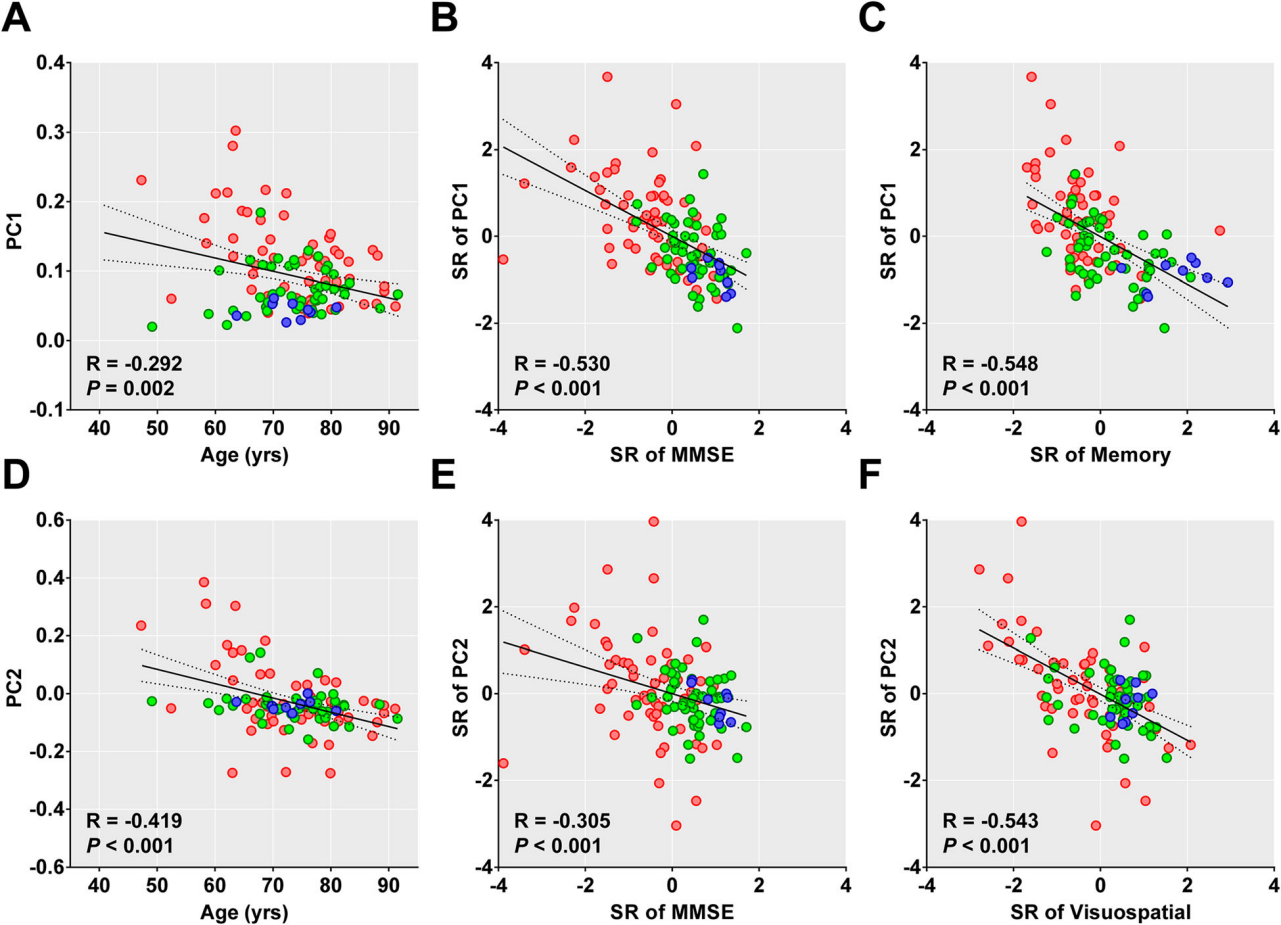
Age-related changes in the PC scores and their correlation with cognitive function in 114 Aβ-positive individuals. The colors of the circles represent individual clinical status (blue = CU, green = MCI, and red = dementia). Pearson’s correlation lines (solid), 95% confidence interval lines (dotted), correlation coefficients (*R*), and *P* values are presented within each plot. PC, principal component; MCI, mild cognitive impairment; CU, cognitively unimpaired; SR, standardized residuals obtained by linear regression analysis with age, years of education, sex, and presence of ApoE ε4 allele as covariates

Both PC1 and PC2 scores decreased with advancing age; however, aging was better reflected by the PC2 score (Fig. 3a, d). Both PC scores decreased with cognition worsening except for the PC2 score with CDR-SB (Additional file 1: Table S2). There was a generally stronger correlation with PC1 than with PC2 scores; however, the visuospatial function showed strongest correlation with the PC2 scores (Fig. 3 and Additional file 1: Table S2). PVE-uncorrected data showed similar PC patterns (Additional file 1: Fig. S2) and results (Additional file 1: Fig. S3 and S4).

We also tested above two different reference sets. Even with the different sets of reference group (114 Aβ-positive participants and 49 Aβ-positive MCI patients), there was no clear difference between the patterns of PCs obtained with the different reference sets (Additional file 1: Fig. S5, S6, S7, and S8). However, the reference set with 56 Aβ-positive AD dementia was best for reflecting global cortical tau burden and global cognition.

### Principal components and tau accumulation rate

**Fig. 4:**
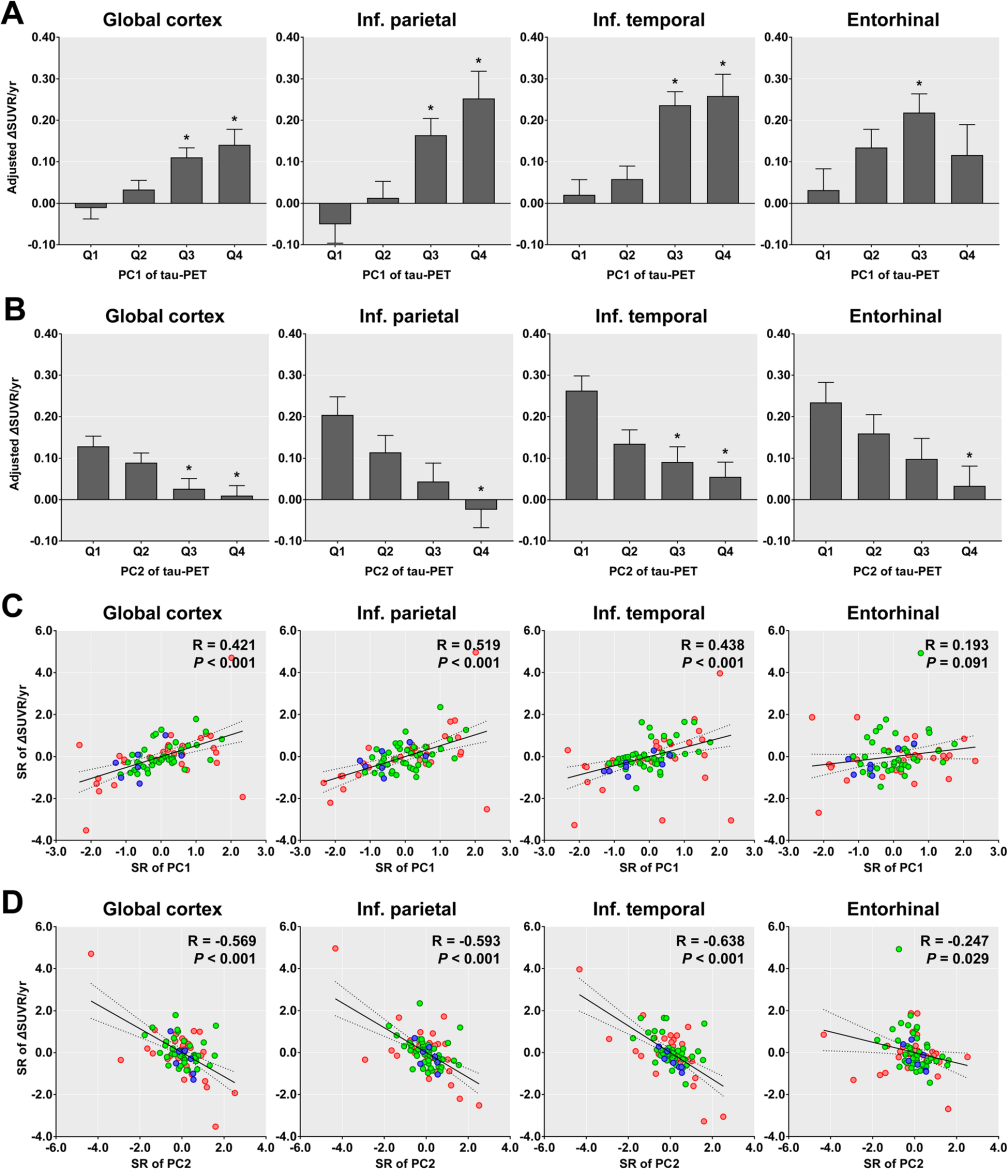
Annual changes in SUVR and expression of two major PCs in 78 Aβ-positive individuals who completed follow-up tau PET. Annual changes adjusted by age, years of education, sex, presence of ApoE ε4 allele, and baseline global cortical SUVR values are presented for each quartile group of PC1 (**a**) and PC2 scores (**b**) obtained from baseline tau PET. The standard residuals for annual changes and those for the two PCs after adjusting for age, years of education, sex, presence of ApoE ε4 allele, and baseline global cortical SUVR values are plotted in **c** and **d**. Asterisks represent a significant difference in the SUVR values between the first and other quartile groups after correction for multiple comparisons. Error bars represent the standard error of estimated means. The colors of the circles represent individual clinical status (blue = CU, green = MCI, and red = AD). Pearson’s correlation lines (solid), 95% confidence interval lines (dotted), correlation coefficients (*R*), and *P* values are presented within each plot. PC, principal component; Qn, quartiles

Among 78 Aβ-positive individuals who underwent follow-up tau PET assessments, annual changes in the SUVR values showed an increase tendency in the baseline PC1 quartile groups and decrease tendency in the baseline PC2 quartile groups (Fig. 4a, b). In the entorhinal cortex, the annual change in SUVR was exceptionally lower in the fourth than in the third quartile group for baseline PC1 scores. Similarly, the annual changes in SUVR correlated with the baseline PC1 and PC2 scores, except for the correlation with the baseline PC1 score in the entorhinal cortex (Fig. 4c, d).

**Fig. 5:**
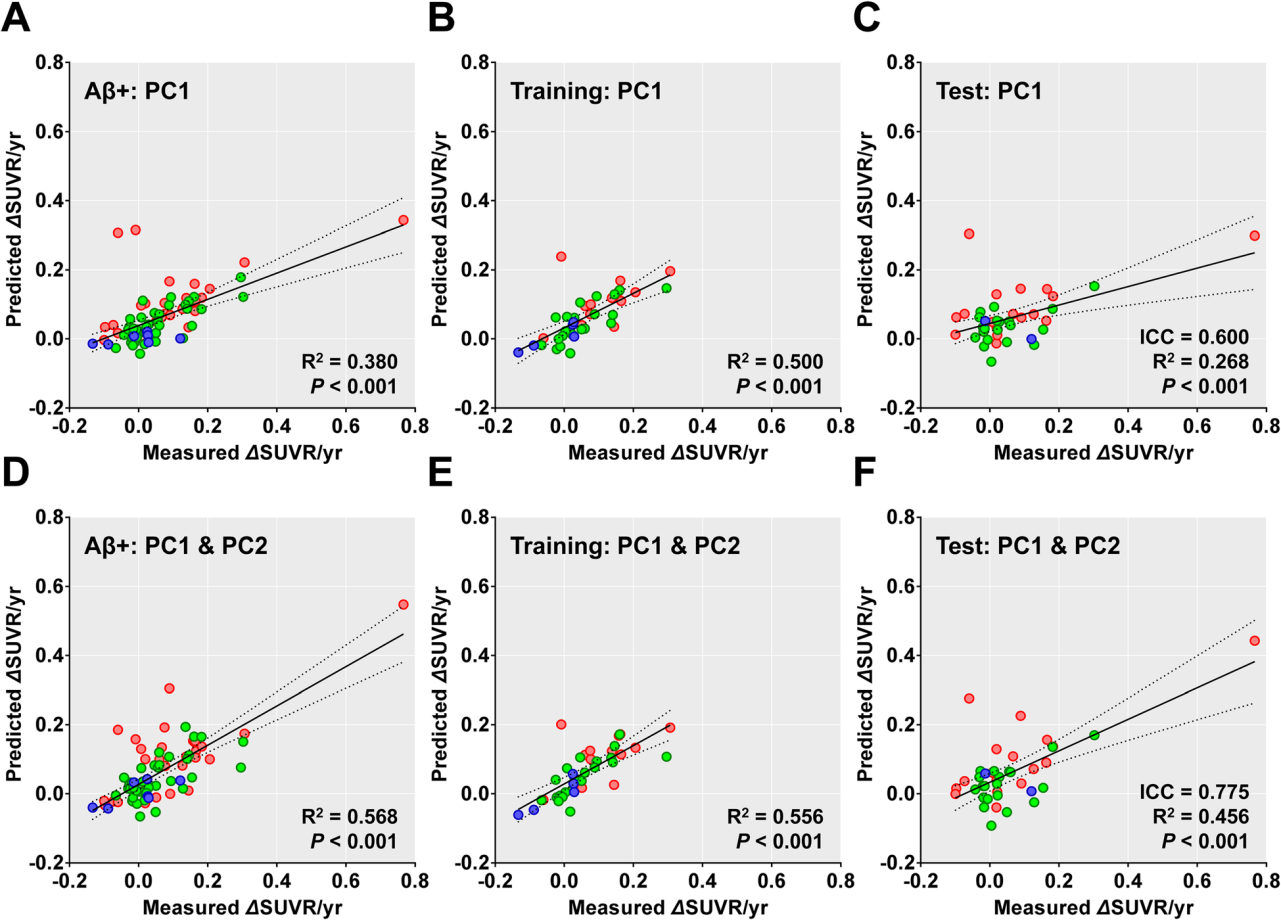
Prediction of annual changes in global cortical SUVR values by baseline PC scores in 78 Aβ-positive individuals who completed follow-up tau PET. The *X*-axes represent measured annual changes based on baseline and follow-up tau PET scans. *Y*-axes represent changes predicted by PC1 scores (**a**–**c**) and both PC1 and PC2 scores (**d**–**f**) after adjustment for age, years of education, sex, and presence of ApoE ε4 allele. Plots for all 78 Aβ-positive individuals (**a**, **d**), 39 individuals in the training set (**b**, **e**), and 39 individuals in the test set (**c**, **f**). Linear regression lines (solid), 95% confidence interval lines (dotted), intraclass correlation coefficient (ICC), *R*^2^, and *P* values are presented within each plot. PC, principal component

A linear regression model that included baseline PC1 scores and demographic data (age, years of education, sex, and presence of ApoE ε4 allele) as independent variables predicted the annual changes in the global cortical SUVR values (*R*^2^ = 0.380, *P* < 0.001) better than the model that included baseline global cortical SUVR (*R*^2^ = 0.237, *P* < 0.001) (Fig. 5a). The model that included both baseline PC1 and PC2 scores had better performance than the model that only included the PC1 score (*R*^2^ = 0.568, *P* < 0.001) (Fig. 5d). We established a regression model for a training set with 39 Aβ-positive individuals and its parameters were applied to the data of the remaining 39 individuals in the test set. The performance of the model including baseline PC1 scores in predicting annual changes was moderate (ICC = 0.600, *R*^2^ = 0.268, *P* < 0.001; Fig. 5b, c). As expected, the model that included both PC scores performed better than the model that only included the PC1 scores (ICC = 0.775, *R*^2^ = 0.456, *P* < 0.001; Fig. 5e, f).

## Discussion

In this study, we found two major Aβ-positive AD dementia-specific PCs of baseline tau PET. PC1 corresponded to the well-known tau distribution and spreading pattern while the two PC2 extremes reflected the temporal or parietal predominance of the tau distribution. PC1 expression was more positively correlated with the global cortical tau burden and negatively correlated with global cognition impairment, compared with PC2 expression. Contrastingly, PC2 expression was more negatively correlated with advancing age and visuospatial and attention function deterioration, compared with PC1 expression. The tau accumulation rate increased with the increase of PC1 expression (individuals with higher tau stage) and with the decrease of PC2 expression (individuals more with parietal predominance). A regression model that combined these two PC scores could predict longitudinal changes in the global cortical tau burden.

Tau pathology first appears in the transentorhinal cortex, with initial spreading toward neighboring medial temporal regions. Subsequently, it spreads to the association neocortex and finally involves the primary cortices [20]. This hierarchical upward spreading pattern of tau pathology has been replicated by in vivo tau PET studies [3, 21]. Moreover, the severity and temporal progression of cognitive dysfunction have been reported to be closely related to the cross-sectional tau burden and longitudinal changes in tau burden measured by tau PET, respectively [4–6, 22]. Therefore, tau PET is an imaging biomarker for the AD stage and progression. In our study, PC1 expression reflected the global tau burden; moreover, the extent of increased ^18^F-flortaucipir uptake in each PC1 quartile group reflected advanced tau stages. Additionally, the global cognition and the functions of five cognitive domains worsened with advancing PC1 expression. These findings indicate that PC1 expression could be a biomarker for the disease stage.

Notably, there was a divergent pattern in PC2 expression in individuals with a high tau burden. The first quartile group for PC2 showed significantly increased ^18^F-flortaucipir uptake predominantly in the medial and lateral temporal cortices. On the other hand, the fourth quartile group showed increased uptake predominantly in the parietal cortex with relative sparing of the medial temporal cortex. These two extremes of ^18^F-flortaucipir uptake patterns are consistent with the distribution patterns of tau pathology and volume atrophy in the typical and hippocampal-sparing AD subtypes [23, 24]. Compared to the typical AD subtype, the hippocampal-sparing AD subtypes, which are characterized by greater pathology (or atrophy) in the diffuse neocortex, exhibit a younger age at onset, greater non-memory function impairment, lower ApoE ε4 genotype frequency, and faster cognitive decline [23–26]. As expected by the age-related decrease in PC2 expression, these two extremes of uptake patterns resemble those found in late- and early-onset AD (LOAD vs. EOAD), specifically the temporal predominance pattern of tau accumulation in LOAD and parietal predominance pattern of tau accumulation in EOAD patients. Similar to the clinical characteristics of EOAD involving more prominent non-memory function than memory function impairment [27–29], as well as worsened visuospatial function with increased tau burden in the parieto-occipital cortex [30], we found a negative correlation of visuospatial and attention function with PC2 expression. Therefore, PC2 expression could be a surrogate marker for AD subtypes.

We previously reported that the baseline tau burden predicted the amount of additional tau accumulation, even in Aβ-positive individuals [6]. Similarly, in this study, we found that baseline PC1 expression was correlated with the tau accumulation rate in the global, inferior parietal, and inferior temporal cortices even after adjustment for the baseline global tau burden. There was a tendency of increased tau accumulation rate in the entorhinal cortex with baseline PC1 expression; however, there was an attenuated tau accumulation rate in the entorhinal cortex in the fourth quartile group of PC1 expression. This could be explained either by possible attenuation of active tau accumulation in regions involved in early Braak’s stage or by the mixture of the individuals with parietal predominance.

Patients with the hippocampal-sparing AD subtype or EOAD have been reported to exhibit faster progression of cognitive impairment and volume atrophy than those with the typical AD subtype or LOAD [23–26, 31]. In this study, except for memory function, estimated annual changes in cognitive test performances were generally greater in the fourth (parietal predominance) of PC2 expression when compared to the first (temporal predominance) quartile group. However, there was no statistical difference between the quartile groups (Additional file 1: Fig. S3). Therefore, we may expect higher tau accumulation rate in individuals with higher PC2 expression. However, contrary to our expectation, tau accumulation rate decreased with increasing baseline PC2 expression after adjusting for demographic variables and baseline global tau burden. Even after only adjusting for demographic variables, the first quartile group showed a significantly higher tau accumulation rate in the global cortex than the fourth quartile group.

Pathological tau protein can be transferred to distant areas through anatomically connected networks via prion-like cell-to-cell transmission [32–36]. In vitro or in vivo studies have reported tau protein propagation through the axon either by anterograde or retrograde transport [33, 37]. However, a postmortem study suggested seeding of pathological tau protein mainly through the anterograde “top-down” connectivity by demonstrating sequential involvement of tau pathology within the cortical neurons from dendrites to axon [38]. Based on this theory, the parietal dominance type of tau accumulation could be closely related to the highest stage of disease progression and therefore has less residual potential for “top-down” transmission than the typical temporal predominance type. The observed low tau accumulation rate observed in individuals with parietal predominance of tau accumulation could be attributed to the limited cortical area for further tau propagation, as well as the early saturation of tau accumulation in regions with a high tau burden.

Since the expression of the two PCs reflected different baseline characteristics and longitudinal changes in tau, we could establish a prediction model for longitudinal tau accumulation. Despite unsatisfactory *R*^2^ and ICC values in the test set for the model that included both PC1 and PC2 scores for predicting the amount of additional tau accumulation, this model was superior to the model that simply included the global cortical SUVR value or just the PCI scores.

### Limitations of study

Our study was primarily limited by the lack of external validation for replicating our findings regarding Aβ-positive AD dementia-specific PC expressions. Moreover, there was a relatively small number of Aβ-positive individuals who completed follow-up tau PET assessments. Further, we did not enroll patients with advanced disease.

## Conclusions

This study shows that PCA of tau PET could be useful for obtaining image-derived biomarkers for disease progression and AD subtypes. Combining the PC expressions could allow the prediction of further tau accumulation.

## Supplementary information

**Additional file 1: Table S1.** Baseline ^18^F-flortaucipir SUVR values for each quartile group of PC2 scores in the 114 Aβ-positive individuals. **Table S2.** Partial correlation analysis between the PC scores and cognitive function in 114 Aβ-positive individuals. PC1 and PC2 scores correlated with the decline of cognitive functions after adjusting for age, years of education, gender, and presence of ApoE ε4 allele. **Table S3.** Estimated annual changes in cognitive test performances after adjusting for demographic variables in each quartile group of PC2 scores. **Table S4.** Correlation between the Aβ-positive AD dementia-specific PC scores and regional Aβ burden. **Fig. S1.** Voxel-based comparisons of tau PET between the 87 Aβ-negative cognitively unimpaired individuals and each quartile group for the expression of Aβ-positive AD dementia-specific PCs. **Fig. S2.** Voxel-wise maps for Aβ-positive AD dementia-specific PCs of tau PET uncorrected for partial volume effect and group-wise averaged tau PET images for each quartile of PC scores in 114 Aβ-positive individuals. **Fig. S3.** Principal components of tau PET uncorrected for partial volume effect in Alzheimer disease. **Fig. S4.** Age-related changes in the PC scores obtained from tau PET images uncorrected for partial volume effect and correlation with cognitive function in 114 Aβ-positive individuals. **Fig. S5.** Voxel-wise maps for PCs of tau PET created with 114 Aβ-positive individuals and group-wise averaged tau PET images for each quartile of PC scores in 114 Aβ-positive individuals. **Fig. S6.** Expression of PCs created with 114 Aβ-positive individuals and global cortical tau burden. **Fig. S7.** Voxel-wise maps for PCs of tau PET created with 49 Aβ-positive MCI patients and group-wise averaged tau PET images for each quartile of PC scores in 114 Aβ-positive individuals. **Fig. S8.** Expression of PCs created with 49 Aβ-positive MCI patients and global cortical tau burden.

## Data Availability

Data generated by this study are available from the corresponding author on reasonable request. The data are not publicly available due to privacy restriction.

## Abbreviations

PCA: Principal component analysis
PCs: Principal components
MCI: Mild cognitive impairment
SUVR: Standardized uptake value ratio
ApoE: Apolipoprotein E
LOAD: Late-onset Alzheimer’s disease
EOAD: Early-onset Alzheimer’s disease
