# Supplementary material for: Principal components of tau positron emission tomography and longitudinal tau accumulation in Alzheimer’s disease
Source: Alzheimers Res Ther. 2020 Sep 23;12:114. doi: 10.1186/s13195-020-00685-4 (PMC7513482; doi:10.1186/s13195-020-00685-4)
Supplement: Supplementary file 1 — Additional file 1: Table S1. Baseline 18F-flortaucipir SUVR values for each quartile group of PC2 scores in the 114 Aβ-positive individuals. Table S2. Partial correlation analysis between the PC scores and cognitive function in 114 Aβ-positive individuals. PC1 and PC2 scores correlated with the decline of cognitive functions after adjusting for age, years of education, gender, and presence of ApoE ε4 allele. Table S3. Estimated annual changes in cognitive test performances after adjusting for demographic variables in each quartile group of PC2 scores. Table S4. Correlation between the Aβ-positive AD dementia-specific PC scores and regional Aβ burden. Fig. S1. Voxel-based comparisons of tau PET between the 87 Aβ-negative cognitively unimpaired individuals and each quartile group for the expression of Aβ-positive AD dementia-specific PCs. Fig. S2. Voxel-wise maps for Aβ-positive AD dementia-specific PCs of tau PET uncorrected for partial volume effect and group-wise averaged tau PET images for each quartile of PC scores in 114 Aβ-positive individuals. Fig. S3. Principal components of tau PET uncorrected for partial volume effect in Alzheimer disease. Fig. S4. Age-related changes in the PC scores obtained from tau PET images uncorrected for partial volume effect and correlation with cognitive function in 114 Aβ-positive individuals. Fig. S5. Voxel-wise maps for PCs of tau PET created with 114 Aβ-positive individuals and group-wise averaged tau PET images for each quartile of PC scores in 114 Aβ-positive individuals. Fig. S6. Expression of PCs created with 114 Aβ-positive individuals and global cortical tau burden. Fig. S7. Voxel-wise maps for PCs of tau PET created with 49 Aβ-positive MCI patients and group-wise averaged tau PET images for each quartile of PC scores in 114 Aβ-positive individuals. Fig. S8. Expression of PCs created with 49 Aβ-positive MCI patients and global cortical tau burden. [file 13195_2020_685_MOESM1_ESM.doc]

**Table S1.** Baseline ^18^F-flortaucipir SUVR values for each quartile group of PC2 scores in the 114 Aβ-positive individuals

|  | **PC2** | | | | | | | | | | | |
| --- | --- | --- | --- | --- | --- | --- | --- | --- | --- | --- | --- | --- |
|  | **Q1** | | | **Q2** | | | **Q3** | | | **Q4** | | |
| **Global cortex** | 2.06 | ± | 0.47 | 1.70 | ± | 0.30**^a^** | 1.65 | ± | 0.33**^a^** | 2.35 | ± | 0.79**^bc^** |
| **Prefrontal** | 2.12 | ± | 0.56 | 1.75 | ± | 0.34**^a^** | 1.73 | ± | 0.47**^a^** | 2.29 | ± | 0.75**^bc^** |
| **Sensorimotor** | 1.55 | ± | 0.24 | 1.44 | ± | 0.16 | 1.46 | ± | 0.20 | 1.91 | ± | 0.54**^abc^** |
| **Sup. parietal** | 1.81 | ± | 0.54 | 1.60 | ± | 0.27 | 1.68 | ± | 0.28 | 2.94 | ± | 1.40**^abc^** |
| **Inf. Parietal** | 2.04 | ± | 0.63 | 1.71 | ± | 0.43 | 1.66 | ± | 0.41 | 2.91 | ± | 1.31**^abc^** |
| **Precuneus** | 2.09 | ± | 0.74 | 1.73 | ± | 0.38 | 1.67 | ± | 0.46 | 3.00 | ± | 1.51**^abc^** |
| **Occipital** | 1.84 | ± | 0.52 | 1.62 | ± | 0.33 | 1.63 | ± | 0.22 | 2.16 | ± | 0.70**^bc^** |
| **Sup. temporal** | 2.02 | ± | 0.65 | 1.59 | ± | 0.28**^a^** | 1.50 | ± | 0.25**^a^** | 2.09 | ± | 0.75**^bc^** |
| **Mid. temporal** | 2.69 | ± | 0.91 | 1.94 | ± | 0.51**^a^** | 1.75 | ± | 0.46**^a^** | 2.51 | ± | 0.95**^bc^** |
| **Inf. temporal** | 2.66 | ± | 0.82 | 1.93 | ± | 0.56**^a^** | 1.76 | ± | 0.46**^a^** | 2.70 | ± | 1.17**^bc^** |
| **Hippocampus** | 2.01 | ± | 0.34 | 1.73 | ± | 0.29**^a^** | 1.58 | ± | 0.35**^a^** | 1.74 | ± | 0.40**^a^** |
| **Entorhinal** | 3.25 | ± | 0.69 | 2.64 | ± | 0.67**^a^** | 2.37 | ± | 0.67**^a^** | 2.54 | ± | 0.72**^a^** |
| **Parahippocampal** | 2.75 | ± | 0.63 | 2.12 | ± | 0.47**^a^** | 1.88 | ± | 0.45**^a^** | 2.35 | ± | 0.71**^c^** |
| **Amygdala** | 2.34 | ± | 0.43 | 1.92 | ± | 0.46**^a^** | 1.74 | ± | 0.51**^a^** | 2.09 | ± | 0.73**^c^** |
| **Ant. cingulate** | 2.16 | ± | 0.77 | 1.65 | ± | 0.22**^a^** | 1.63 | ± | 0.44**^a^** | 1.85 | ± | 0.49 |
| **Post. cingulate** | 2.21 | ± | 0.62 | 1.78 | ± | 0.43**^a^** | 1.69 | ± | 0.51**^a^** | 2.59 | ± | 1.11**^bc^** |
| **Insula** | 2.03 | ± | 0.51 | 1.55 | ± | 0.23**^a^** | 1.49 | ± | 0.37**^a^** | 1.80 | ± | 0.55**^c^** |

^a^*P* < 0.05 for the comparison between the first and the other quartile groups, ^b^*P* < 0.05 for the comparison between the second and the other quartile group, ^a^*P* < 0.05 for the comparison between the third and the other quartile groups

Abbreviations: SUVR: standardized uptake value ratio, PC: principal component, Qn: quartiles

**Table S2.** Partial correlation analysis between the PC scores and cognitive function in 114 Aβ-positive individuals

|  | **PC1** | |  | **PC2** | |
| --- | --- | --- | --- | --- | --- |
|  | **R** | ***P*-value** |  | **R** | ***P*-value** |
| **MMSE** | -0.530 | **< 0.001** |  | -0.305 | **< 0.001** |
| **CDR-SB** | 0.474 | **< 0.001** |  | 0.173 | 0.066 |
| **Total cognition score** | -0.611 | **< 0.001** |  | -0.373 | **< 0.001** |
| **Memory** | -0.548 | **< 0.001** |  | -0.221 | **0.019** |
| **Language** | -0.449 | **< 0.001** |  | -0.286 | **0.002** |
| **Visuospatial** | -0.448 | **< 0.001** |  | -0.543 | **< 0.001** |
| **Frontal/executive** | -0.566 | **< 0.001** |  | -0.277 | **0.003** |
| **Attention** | -0.273 | **0.004** |  | -0.342 | **< 0.001** |

PC1 and PC2 scores correlated with the decline of cognitive functions after adjusting for age, years of education, gender, and presence of ApoE ε4 allele.

Abbreviations: PC: principal component, R: Pearson's correlation coefficient

**Table S3.** Estimated annual changes in cognitive test performances after adjusting for demographic variables in each quartile group of PC2 scores

|  | **PC2** | | | | | | | |
| --- | --- | --- | --- | --- | --- | --- | --- | --- |
|  | **Q1** | | **Q2** | | **Q3** | | **Q4** | |
| **MMSE** | -1.2 | (0.5) | -1.2 | (0.4) | -0.5 | (0.4) | -2.3 | (0.5) |
| **CDR-SB** | 0.8 | (0.3) | 0.6 | (0.3) | 0.5 | (0.3) | 1.1 | (0.3) |
| **Total cognition score** | -21.7 | (3.5) | -23.4 | (3.2) | -14.4 | (3.4) | -23.6 | (3.5) |
| **Memory** | -3.9 | (2.0) | -4.5 | (1.8) | 0.5 | (2.0) | -3.5 | (2.0) |
| **Language** | -0.8 | (0.4) | -0.3 | (0.4) | -0.3 | (0.4) | -1.6 | (0.4) |
| **Visuospatial** | -1.3 | (0.9) | -1.1 | (0.9) | 0.2 | (0.9) | -3.0 | (0.9) |
| **Frontal/executive** | -0.7 | (1.2) | -1.3 | (1.1) | -0.3 | (1.1) | -2.4 | (1.2) |
| **Attention** | -0.7 | (0.3) | -0.3 | (0.2) | -0.1 | (0.3) | -0.7 | (0.3) |

Data are presented as estimated mean (standard error).

Abbreviations: PC: principal component, Qn: quartiles

**Table S4.** Correlation between the Aβ-positive AD dementia-specific PC scores and regional Aβ burden

|  | **All 272 participants** | | | | |  | **114 Aβ-positive participants** | | | | |
| --- | --- | --- | --- | --- | --- | --- | --- | --- | --- | --- | --- |
|  | **PC1** | |  | **PC2** | |  | **PC1** | |  | **PC2** | |
|  | **R** | ***P*** |  | **R** | ***P*** |  | **R** | ***P*** |  | **R** | ***P*** |
| **Global cortex** | 0.657 | **< 0.001^*^** |  | 0.097 | 0.111 |  | 0.403 | **< 0.001^*^** |  | 0.221 | **0.018^*^** |
| **Prefrontal** | 0.632 | **< 0.001^*^** |  | 0.054 | 0.378 |  | 0.352 | **< 0.001^*^** |  | 0.133 | 0.157 |
| **Sensorimotor** | 0.634 | **< 0.001^*^** |  | 0.137 | **0.024** |  | 0.388 | **< 0.001^*^** |  | 0.252 | **0.007^*^** |
| **Sup. parietal** | 0.631 | **< 0.001^*^** |  | 0.162 | **0.007** |  | 0.379 | **< 0.001^*^** |  | 0.318 | **< 0.001^*^** |
| **Inf. Parietal** | 0.664 | **< 0.001^*^** |  | 0.122 | **0.044** |  | 0.431 | **< 0.001^*^** |  | 0.267 | **0.004^*^** |
| **Precuneus** | 0.664 | **< 0.001^*^** |  | 0.109 | 0.073 |  | 0.424 | **< 0.001^*^** |  | 0.249 | **0.008^*^** |
| **Occipital** | 0.617 | **< 0.001^*^** |  | 0.184 | **0.002^*^** |  | 0.330 | **< 0.001^*^** |  | 0.344 | **< 0.001^*^** |
| **Sup. temporal** | 0.667 | **< 0.001^*^** |  | 0.112 | 0.065 |  | 0.419 | **< 0.001^*^** |  | 0.241 | **0.010^*^** |
| **Mid. temporal** | 0.652 | **< 0.001^*^** |  | 0.056 | 0.355 |  | 0.387 | **< 0.001^*^** |  | 0.142 | 0.131 |
| **Inf. temporal** | 0.681 | **< 0.001^*^** |  | 0.111 | 0.068 |  | 0.451 | **< 0.001^*^** |  | 0.246 | **0.008^*^** |
| **Hippocampus** | 0.483 | **< 0.001^*^** |  | -0.018 | 0.769 |  | 0.228 | **0.015^*^** |  | 0.016 | 0.864 |
| **Entorhinal** | 0.353 | **< 0.001^*^** |  | 0.073 | 0.227 |  | 0.051 | 0.591 |  | 0.140 | 0.137 |
| **Parahippocampal** | 0.588 | **< 0.001^*^** |  | 0.021 | 0.735 |  | 0.255 | **0.006^*^** |  | 0.082 | 0.386 |
| **Amygdala** | 0.522 | **< 0.001^*^** |  | 0.014 | 0.814 |  | 0.283 | **0.002^*^** |  | 0.053 | 0.579 |
| **Ant. cingulate** | 0.608 | **< 0.001^*^** |  | 0.022 | 0.722 |  | 0.307 | **< 0.001^*^** |  | 0.073 | 0.438 |
| **Post. cingulate** | 0.648 | **< 0.001^*^** |  | 0.062 | 0.311 |  | 0.374 | **< 0.001^*^** |  | 0.165 | 0.080 |
| **Insula** | 0.631 | **< 0.001^*^** |  | 0.075 | 0.220 |  | 0.347 | **< 0.001^*^** |  | 0.169 | 0.072 |

Asterisks represent regions survived after correcting for region-wise multiple comparisons.


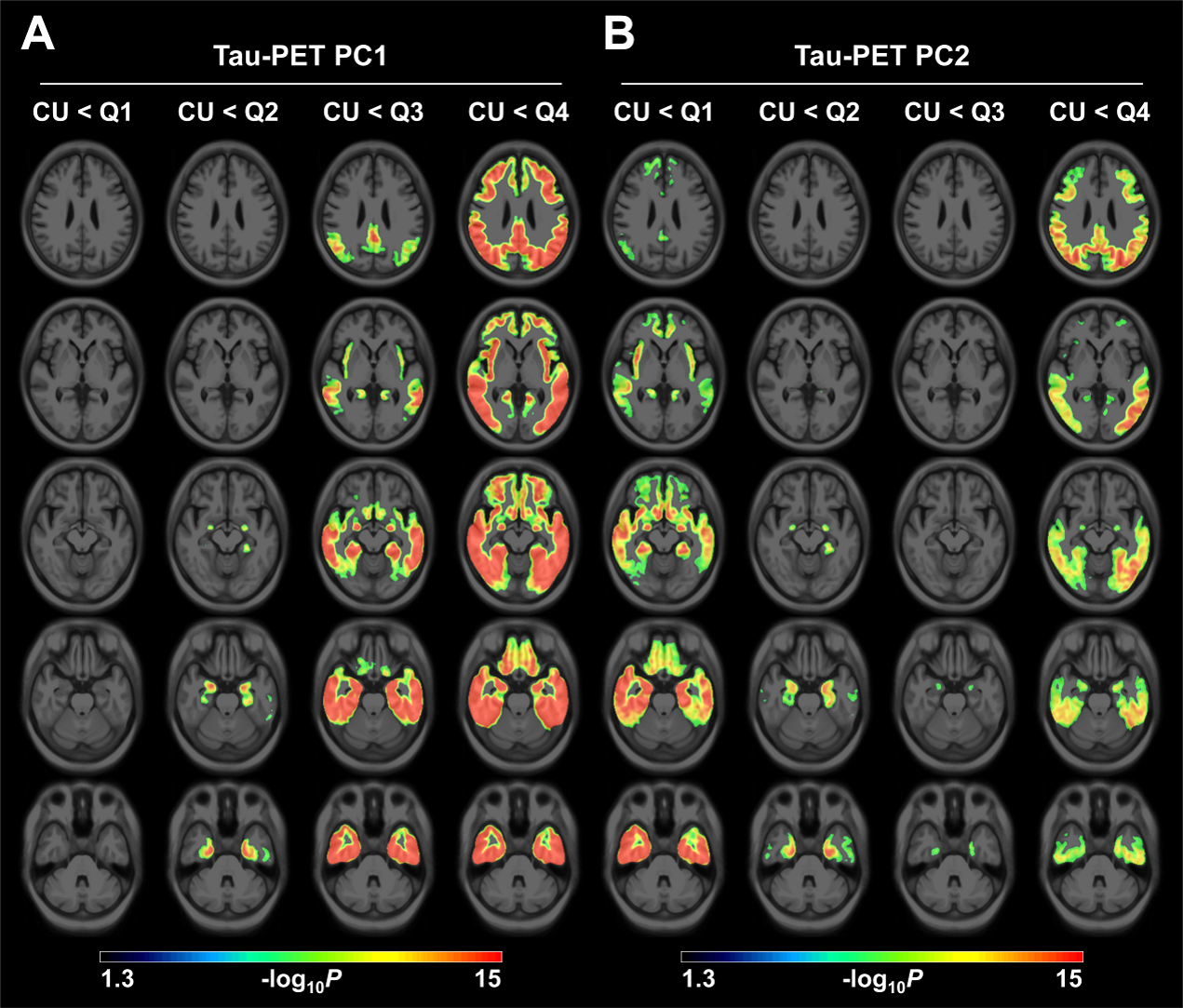


**Fig. S1.** Voxel-based comparisons of tau PET between the 87 Aβ-negative cognitively unimpaired individuals and each quartile group for the expression of Aβ-positive AD dementia-specific PCs

Voxels that remain after family-wise error-corrected *P* < 0.05 are displayed. Color bars represent -log_10_*P*-value for each comparison.

Abbreviations: PC: principal component, AD: Alzheimer disease, SUVR: standardized uptake value ratio, Qn: quartiles


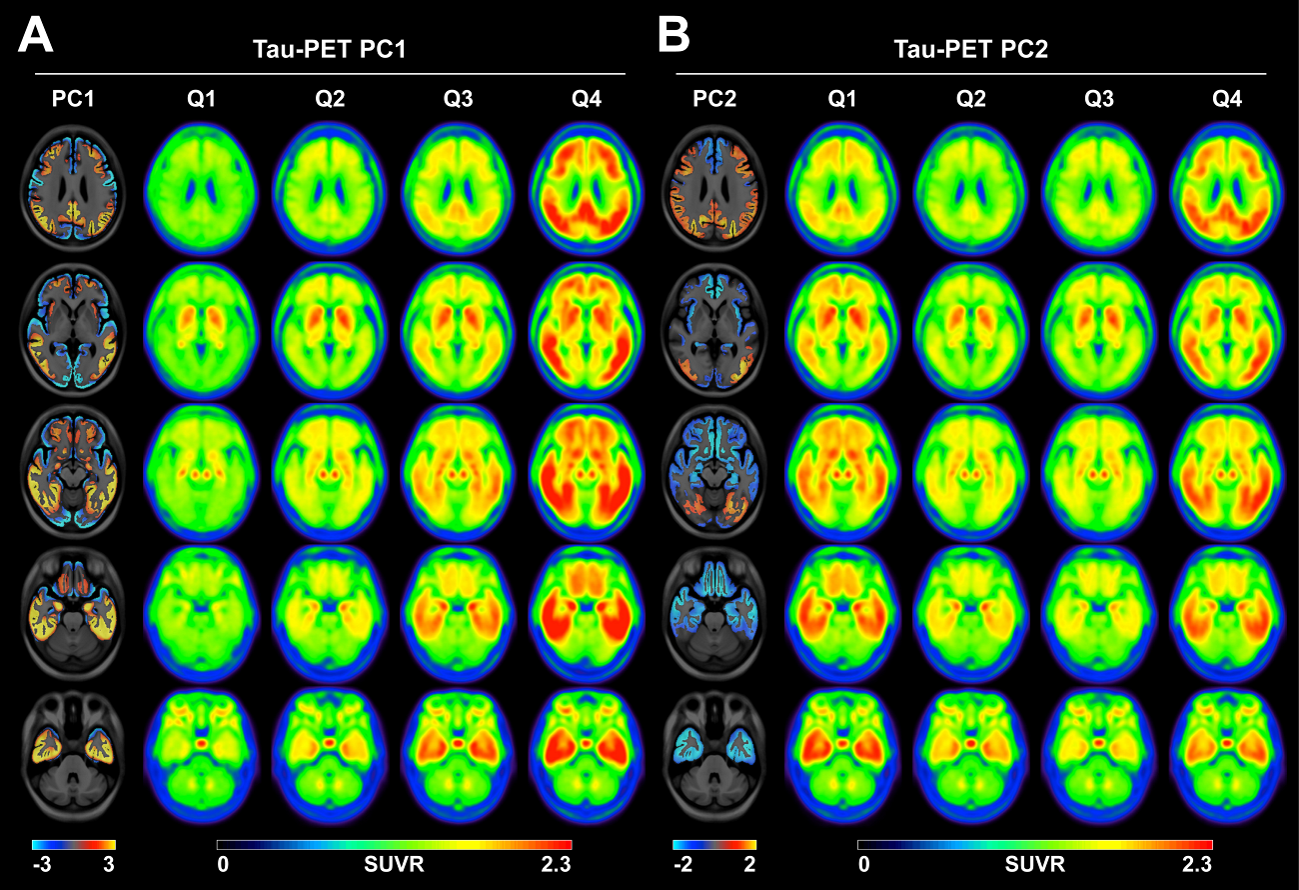


**Fig. S2.** Voxel-wise maps for Aβ-positive AD dementia-specific PCs of tau PET uncorrected for partial volume effect and group-wise averaged tau PET images for each quartile of PC scores in 114 Aβ-positive individuals

There was an increasing trend of the cortical tau burden with the advancement of PC1 quartile groups. The lowest PC2 quartile group (Q1) showed a temporal predominance pattern while the highest PC2 quartile group (Q4) showed a parietal predominance pattern.

The color bars represent PCs (bidirectional cold and hot colors) and SUVR (rainbow color).

Abbreviations: PC: principal component, AD: Alzheimer disease, SUVR: standardized uptake value ratio, Qn: quartiles


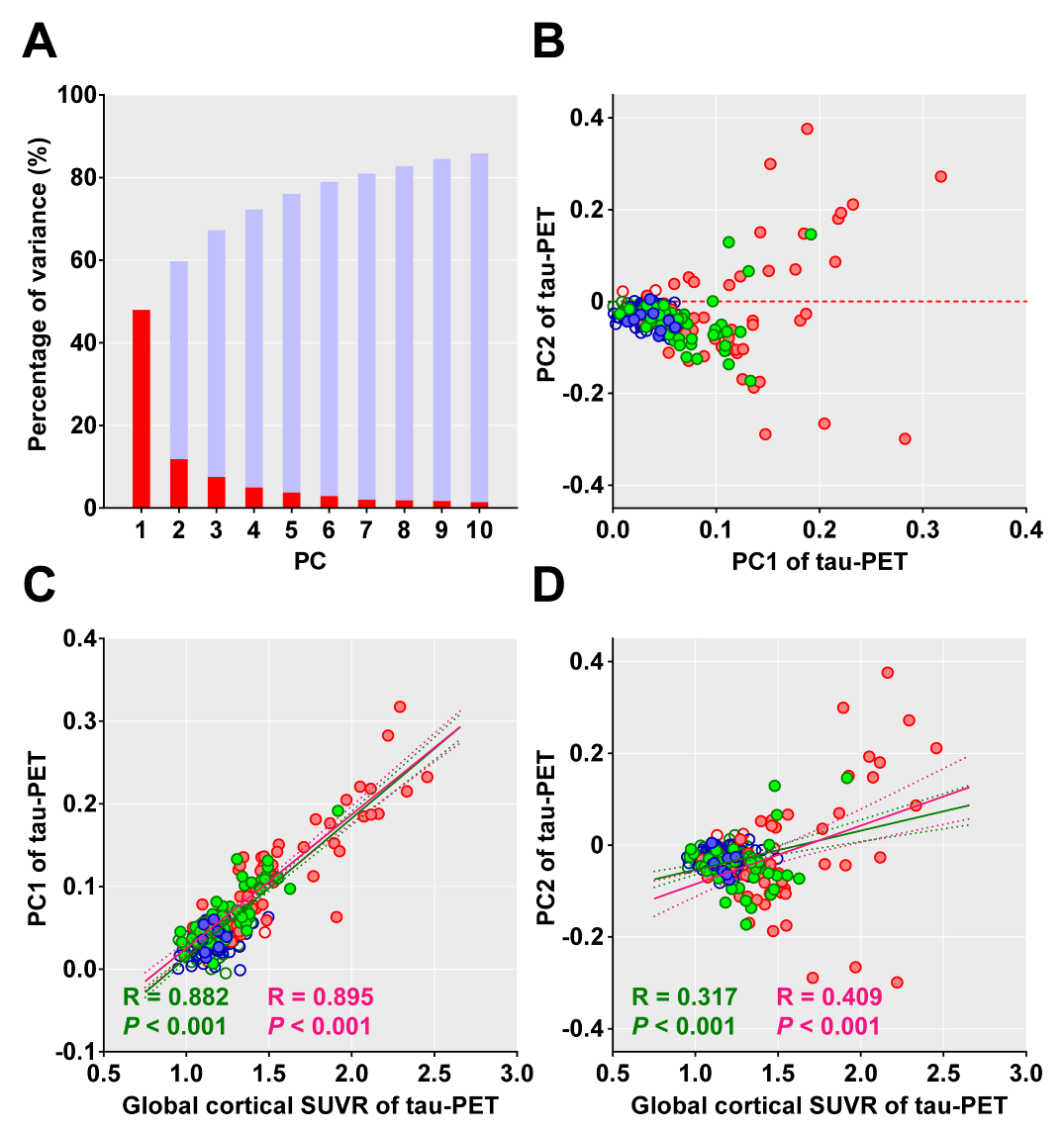


**Fig. S3.** Principal components of tau PET uncorrected for partial volume effect in Alzheimer disease

(A) The percentages of variance explained by the first 10 PCs (red bars) and their cumulative percentages (light blue bars). The first two PCs (PC1 and PC2) contributed to 48% and 12% of the total variance, respectively. (B) Scatter plot of individual scores for PC1 and PC2 expression exhibits divergence of PC2 scores with increasing PC1 scores. (C) The degree of PC1 expression clearly reflects the cortical tau burden measured by global cortical SUVR. (D) PC2 scores were likely to increase with the global cortical SUVR value; however, there was a divergence of PC2 scores in individuals with a higher tau burden.

The colors of the circles represent individual clinical statuses (blue, CU; green, MCI; and red, AD) while closed or open circles represent individuals with or without Aβ positivity, respectively. Pearson’s correlation lines (solid), 95% confidence interval lines (dotted), correlation coefficients (R), and *P*-values are presented in green color (all 272 individuals) or red color (114 Aβ-positive individuals).

Abbreviations: PC: principal component, AD: Alzheimer disease, MCI: mild cognitive impairment, CU: cognitively unimpaired


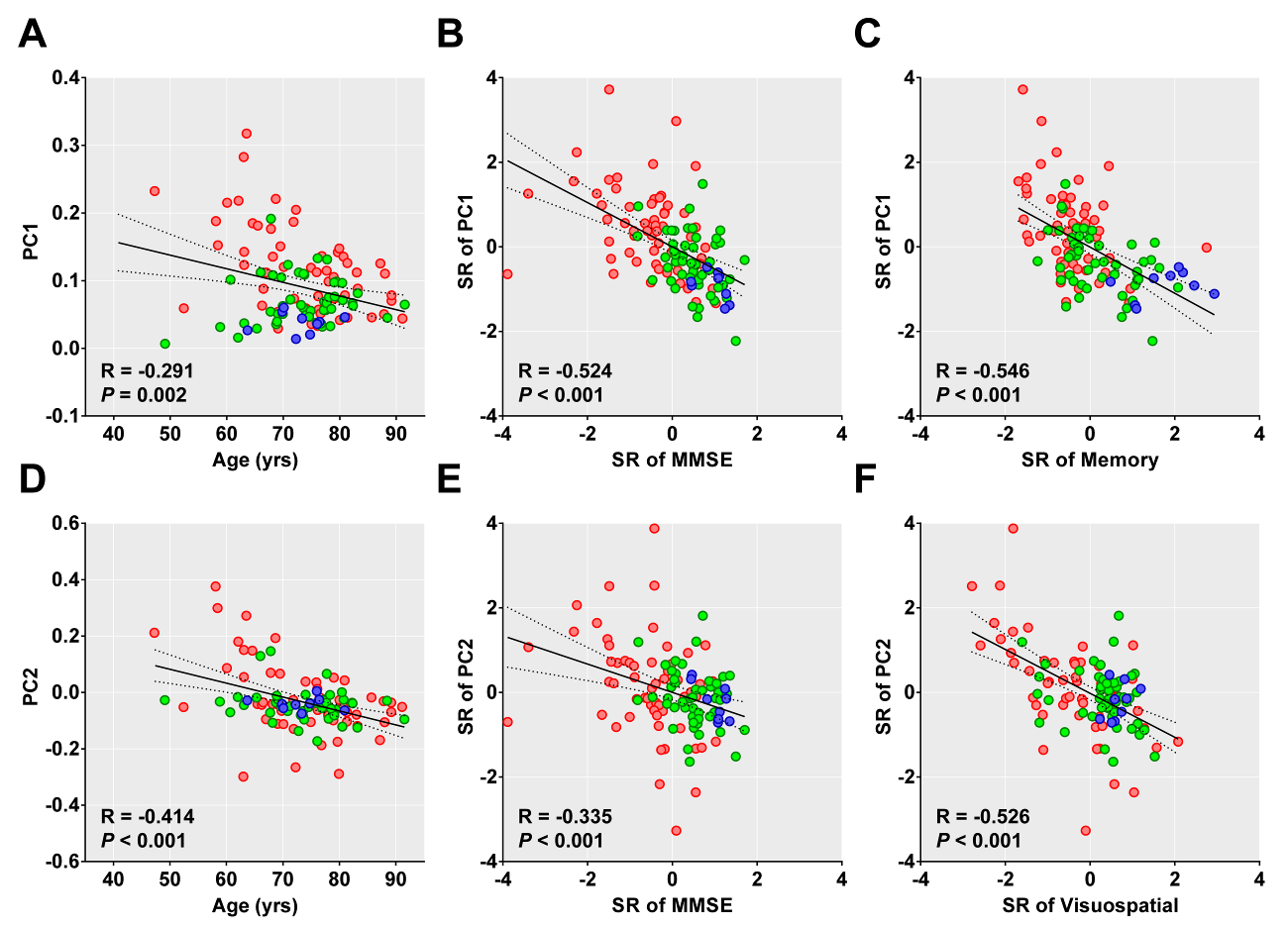


**Fig. S4.** Age-related changes in PC scores obtained from tau PET uncorrected for partial volume effect and correlation with cognitive function in 114 Aβ positive individuals

The colors of the circles represent individual clinical statuses (blue, CU; green, MCI; and red, AD). Pearson’s correlation lines (solid), 95% confidence interval lines (dotted), correlation coefficients (R), and *P*-values are presented within each plot.

Abbreviations: PC: principal component, AD: Alzheimer disease, MCI: mild cognitive impairment, CU: cognitively unimpaired, SR: standardized residuals obtained by linear regression analysis with age, years of education, gender, and presence of ApoE ε4 allele as covariates


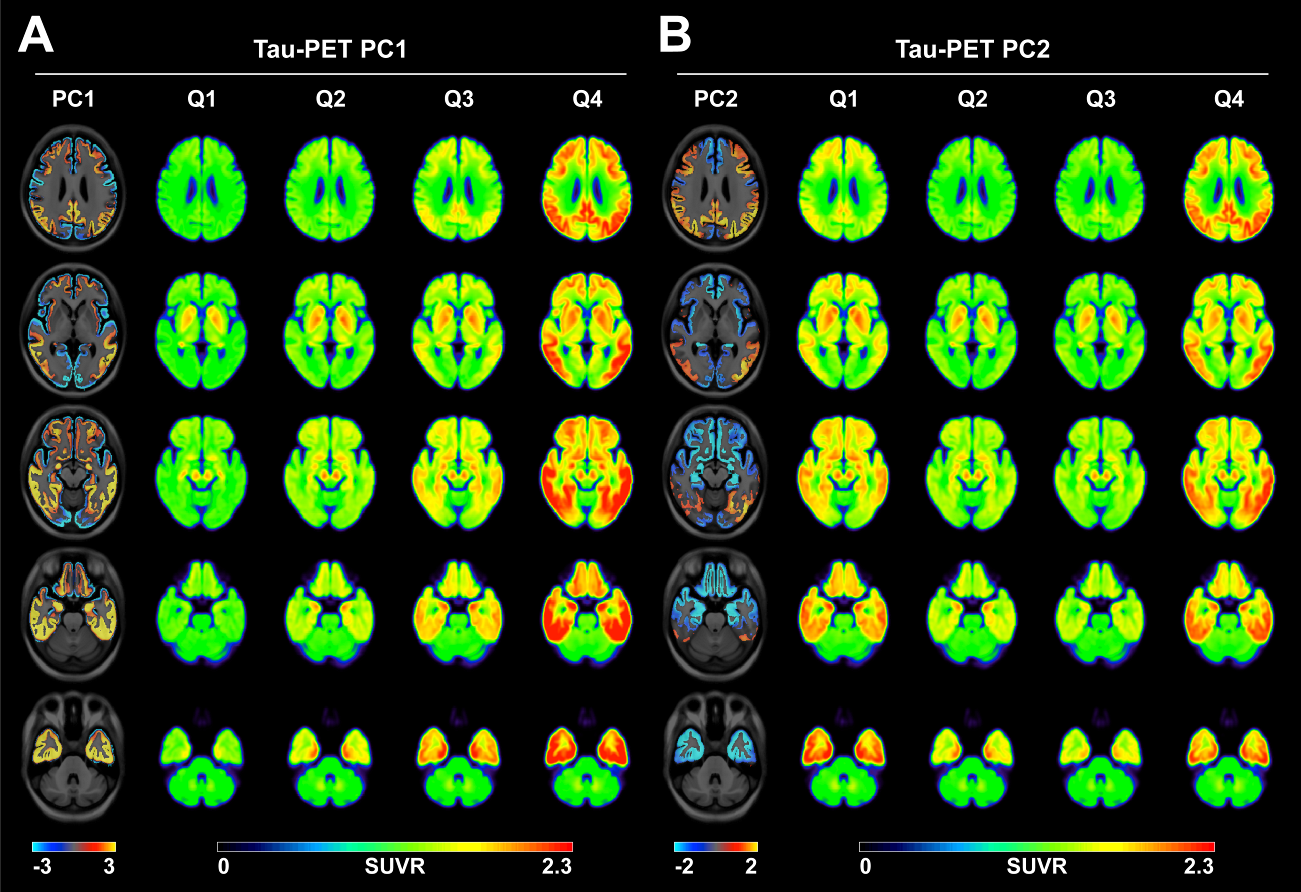


**Fig. S5.** Voxel-wise maps for PCs of tau PET created with 114 Aβ-positive individuals and group-wise averaged tau PET images for each quartile of PC scores in 114 Aβ-positive individuals

The color bars represent PCs (bidirectional cold and hot colors) and SUVR (rainbow color).

Abbreviations: PC: principal component, AD: Alzheimer disease, SUVR: standardized uptake value ratio, Qn: quartiles


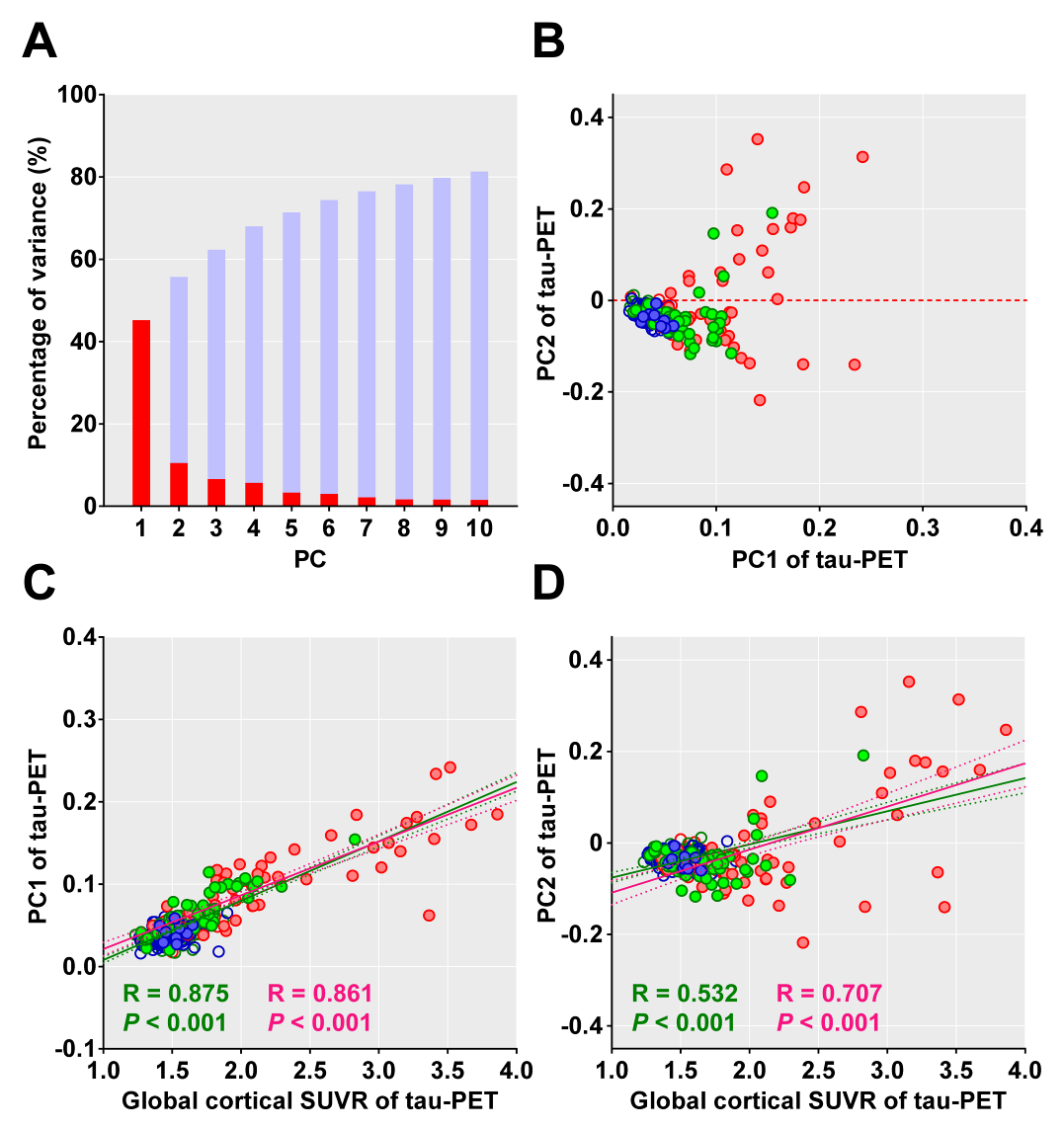


**Fig. S6.** Expression of PCs created with 114 Aβ-positive individuals and global cortical tau burden

(A) The percentages of variance explained by the first 10 PCs (red bars) and their cumulative percentages (light blue bars). (B) Scatter plot of individual scores for PC1 and PC2 expression. (C) The degree of PC1 expression correlate with the cortical tau burden. (D) PC2 scores were likely to increase with the global cortical SUVR value; however, there was a divergence of PC2 scores in individuals with a higher tau burden.

The colors of the circles represent individual clinical statuses (blue, CU; green, MCI; and red, AD) while closed or open circles represent individuals with or without Aβ positivity, respectively. Pearson’s correlation lines (solid), 95% confidence interval lines (dotted), correlation coefficients (R), and *P*-values are presented in green color (all 272 individuals) or red color (114 Aβ-positive individuals).

Abbreviations: PC: principal component, AD: Alzheimer disease, MCI: mild cognitive impairment, CU: cognitively unimpaired


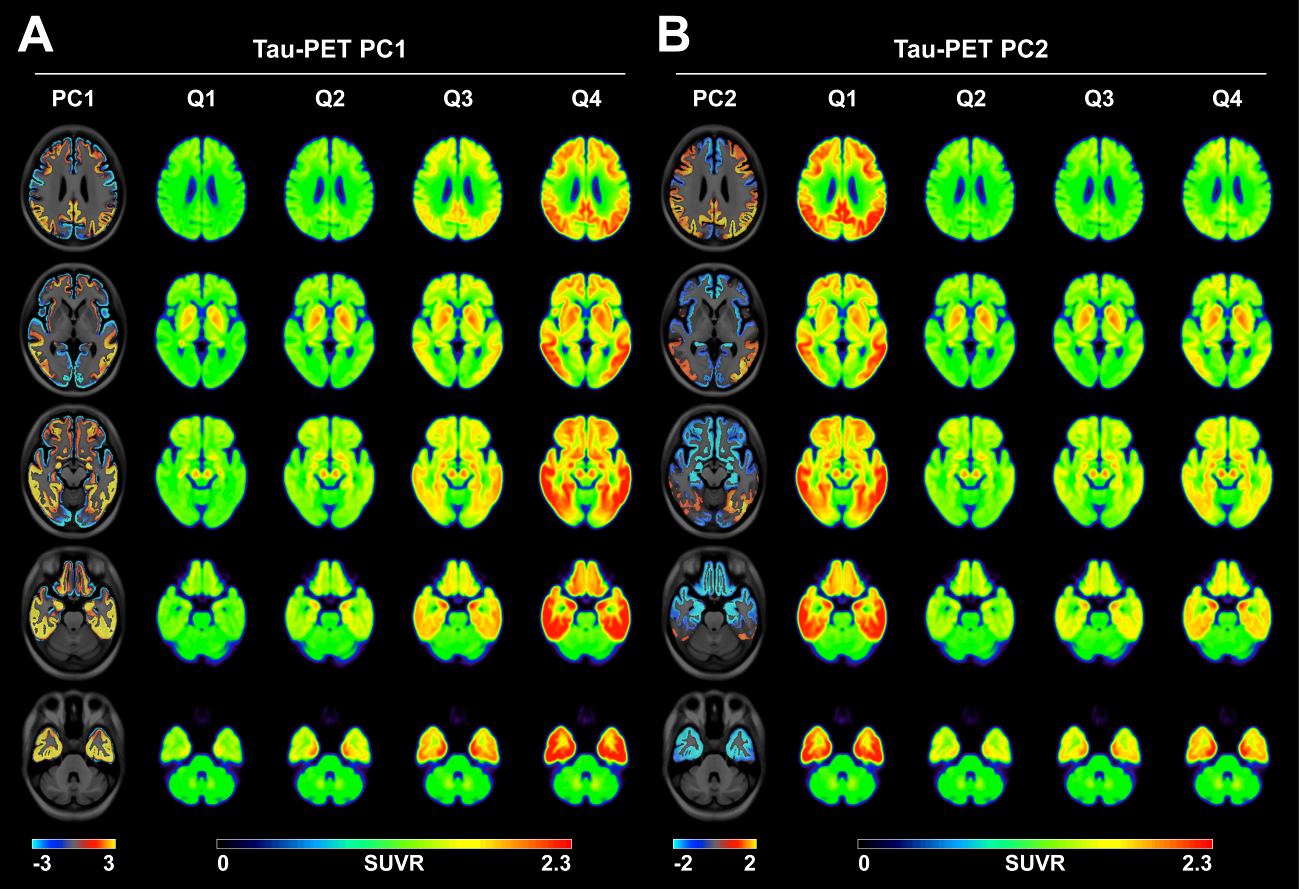


**Fig. S7.** Voxel-wise maps for PCs of tau PET created with 49 Aβ-positive MCI patients and group-wise averaged tau PET images for each quartile of PC scores in 114 Aβ-positive individuals

The color bars represent PCs (bidirectional cold and hot colors) and SUVR (rainbow color).

Abbreviations: PC: principal component, AD: Alzheimer disease, SUVR: standardized uptake value ratio, Qn: quartiles


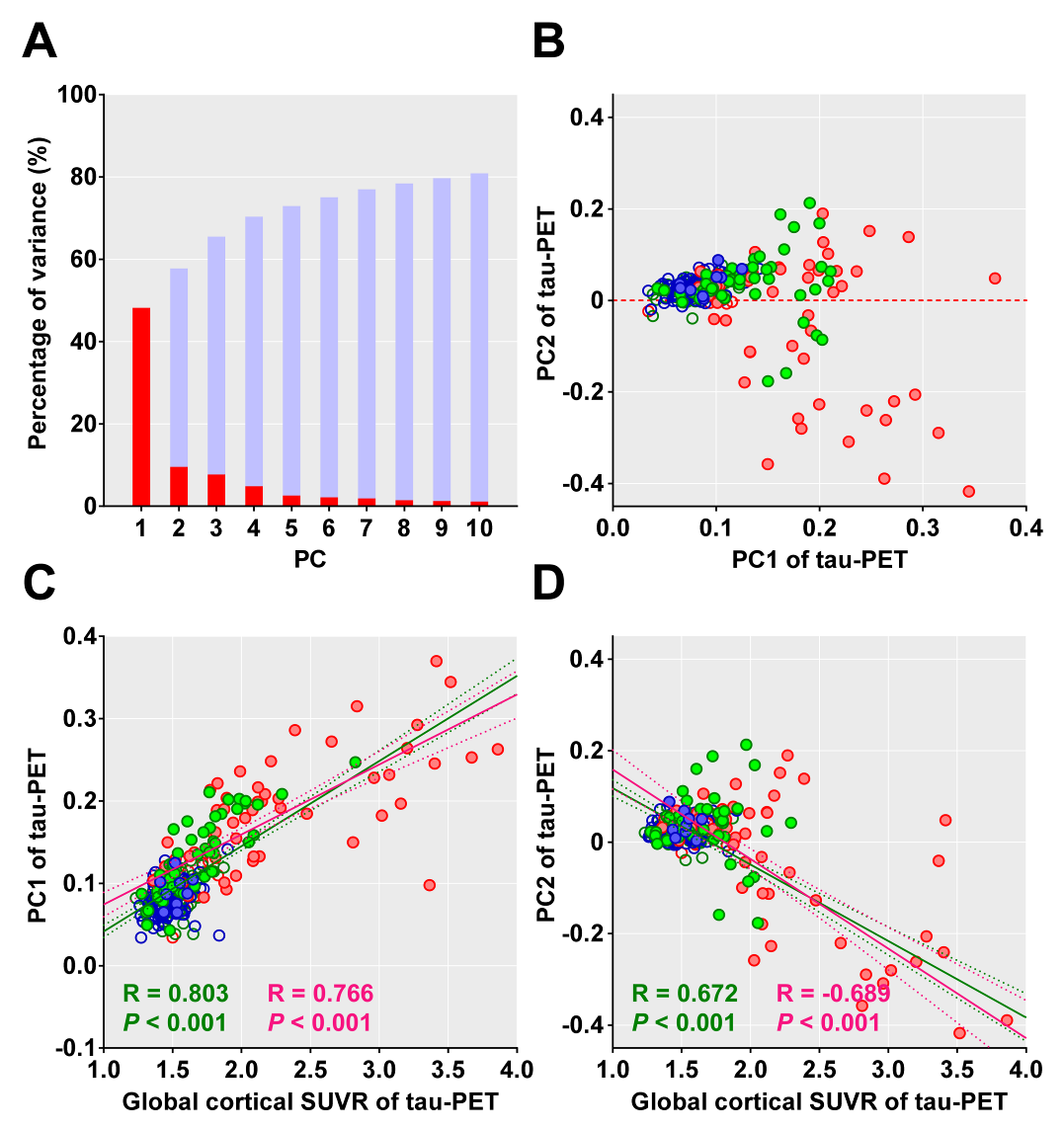


**Fig. S8.** Expression of PCs created with 49 Aβ-positive MCI patients and global cortical tau burden

(A) The percentages of variance explained by the first 10 PCs (red bars) and their cumulative percentages (light blue bars). (B) Scatter plot of individual scores for PC1 and PC2 expression. (C) The degree of PC1 expression correlate with the cortical tau burden. (D) PC2 scores were likely to increase with the global cortical SUVR value; however, there was a divergence of PC2 scores in individuals with a higher tau burden.

The colors of the circles represent individual clinical statuses (blue, CU; green, MCI; and red, AD) while closed or open circles represent individuals with or without Aβ positivity, respectively. Pearson’s correlation lines (solid), 95% confidence interval lines (dotted), correlation coefficients (R), and *P*-values are presented in green color (all 272 individuals) or red color (114 Aβ-positive individuals).

Abbreviations: PC: principal component, AD: Alzheimer disease, MCI: mild cognitive impairment, CU: cognitively unimpaired
